# ‘Ultrasound Examination’ of the Musculoskeletal System: Bibliometric/Visualized Analyses on the Terminology (Change)

**DOI:** 10.3390/tomography9010028

**Published:** 2023-02-02

**Authors:** Carmelo Pirri, Nina Pirri, Carla Stecco, Veronica Macchi, Andrea Porzionato, Raffaele De Caro, Levent Özçakar

**Affiliations:** 1 Department of Neurosciences, Institute of Human Anatomy, University of Padova, 35121 Padua, Italy; 2 Department of Medicine—DIMED, School of Radiology, Radiology Institute, University of Padua, 35122 Padova, Italy; 3 Department of Physical and Rehabilitation Medicine, Hacettepe University Medical School, 06100 Ankara, Turkey

**Keywords:** musculoskeletal, ultrasonography, sono-palpation, dynamic imaging, anatomy, nerve

## Abstract

Ultrasound imaging of the musculoskeletal system is paramount for physicians of different specialties. In recent years, its use has become the extension of physical examinations like using a “magnifying glass”. Likewise, the eventual concept has naturally and spontaneously evolved to a “fusion” of classical physical examination and static/dynamic ultrasound imaging of the musculoskeletal system. In this regard, we deem it important to explore the current use/awareness regarding ‘ultrasound examination’, and to better provide insight into understanding future research spots in this field. Accordingly, this study aimed to search the global/research status of ‘ultrasound examination’ of the musculoskeletal system based on bibliometric and visualized analysis.

## 1. Introduction

Ultrasound (US) imaging has long been used in the diagnosis of various musculoskeletal disorders. Real-time US guidance is also fundamental for precise therapeutic interventions [1,2,3]. Nowadays, owing to its several advantages (convenient, patient/physician-friendly, providing dynamic imaging and comparison, etc.), the usage of US imaging in the daily practice of musculoskeletal medicine has resulted in the merging of classical physical examination with static/dynamic US imaging, becoming a single integrated assessment procedure whereby US plays the role of the ‘magnifying glass’ [4,5,6]. Additionally, its usefulness in reducing the cost, radiation exposure and waiting period in the clinical setting has also been reported [7].

Many researchers have suggested ways to use the US probe to perform various steps of physical examination [4,8,9,10]. For instance, sono-palpation (or sono-Tinel in case of nerve imaging) is sometimes second to none for prompting the exact cause of a painful complaint while imaging/examining the patient [11,12]. Further, with the possibility of physician–patient interaction during the examination, ‘self-palpation’ might even be used [13]. As a side note, the understanding of how different tissues and anatomical structures move/interact with each other is fundamental to holistically interpret the (dys)function and pathology of patients [4]. Likewise, certain special tests can readily be performed under US imaging as well [14]. 

Bibliometrics can help researchers to easily and quantitatively/qualitatively grasp the scientific output and research trend related to this emerging tool, in different fields [15,16,17]. The information displayed is often more robust when combined with visual analysis and has been used in several medical research domains [18,19,20,21]. Therefore, the purpose of this study was to use bibliometrics and visualization of bibliometric networks for discussing the clinical awareness/use of ‘US examination’ in different clinical/research scenarios of musculoskeletal medicine.

## 2. Materials and Methods

**Figure 1:**
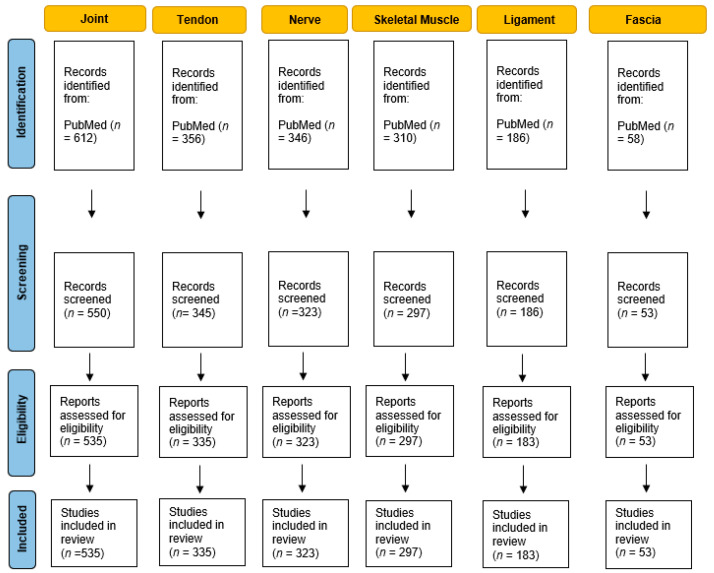
Study flow diagram shows bibliometric analysis protocol for different anatomical musculoskeletal structures and ultrasound examination.

In December 2022, all papers published between 1 January 1960 and 30 December 2022, were retrieved from PubMed, according to the search strategy set for the topic: (“Ultrasound Examination” AND “Joint”) OR (“Ultrasound Examination” AND “Tendon”) OR (“Ultrasound Examination” AND “Nerve”) OR (“Ultrasound Examination” AND “Skeletal Muscle”) OR (“Ultrasound Examination” AND “Ligament”) OR (“Ultrasound Examination” AND “Fascia”). Every type of document was included. A total of 1867 papers were retrieved. The selection criteria for the screening were: (1) English language; (2) timespan: between 1 January 1960 and 30 December 202; (3) articles that exclusively targeted the topic with the terminology ‘Ultrasound examination’ for different anatomical musculoskeletal structures. After the manual screening, only the papers related to the research topic (N = 1726) were included and data on the following variables were extracted: title, publication year, country or region, journal, references and keywords (Figure 1). 

### Analysis of Results

All data were imported into Microsoft Excel 2010 for collation. Citation features were analyzed using Scopus Analyzer, Microsoft Excel and VOSviewer. The H-index by Scopus (https://www.scopus.com/search/form.uri?display=basic#basic) (accessed on 21 December 2022) was used to estimate the importance or impact of citations obtained from PubMed papers. Microsoft Excel 2010 was used to show the number of selected publications per year, country or region and journals. VOSviewer software was used to create a visual representation of keywords. The keyword map involved only keywords that occurred in the title and abstract at least 5 times under binary counting. Keywords with the highest relevance score were used to create the keywords maps for network visualization. The algorithm was designed to ensure that keywords that co-occurred more frequently had larger bubbles and keywords that have a high similarity are located close to each other. 

## 3. Results

### 3.1. Annual Distribution of Publications 

**Figure 2:**
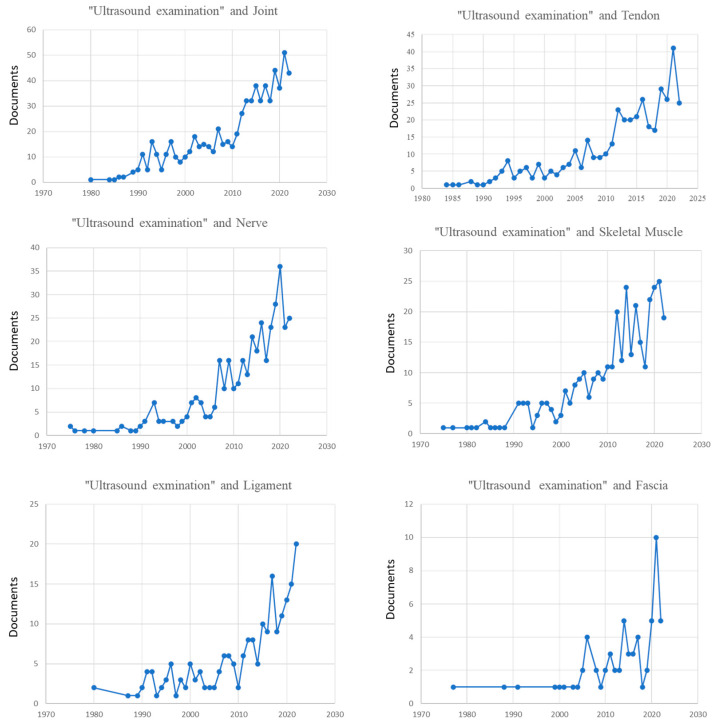
Number of papers published on ultrasound examination and different structures of the musculoskeletal system.

Over the last 22 years, the number of papers on US examination of different musculoskeletal structures has increased (Figure 2). 

The number of publications significantly increased after 2010. In the period of time between 1990 to 2010, the number of papers was growing without a real surge in the number of publications.

### 3.2. Countries or Regions 

**Figure 3:**
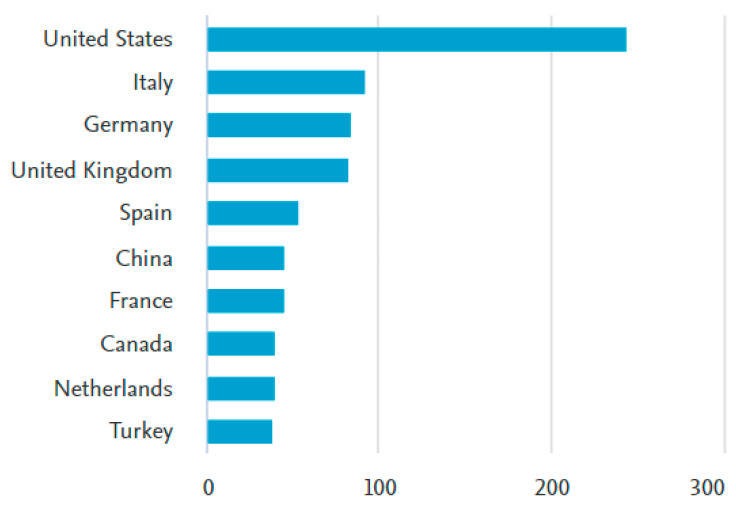
Top 10 countries or regions reporting studies on musculoskeletal system and ultrasound examination. Total studies of the top 10 countries = 753 papers.

A total of 1726 papers were published in 79 countries or regions. The top 10 regions with greatest number of publications included the United States of America (*n* = 243), Italy (*n* = 91), Germany (*n* = 83), the United Kingdom (*n* = 81), Spain (*n* = 52), China (*n* = 44), France (*n* = 44), Canada (*n* = 39), Netherlands (*n* = 39) and Turkey (*n* = 37). (Figure 3)

### 3.3. Journals

Scopus Analyzer, Microsoft Excel and VOSviewer were used to analyze the citation sources, i.e., identifying the journals with the maximum use of this terminology (Table 1). 

### 3.4. Co-Occurrence Analysis

**Figure 4:**
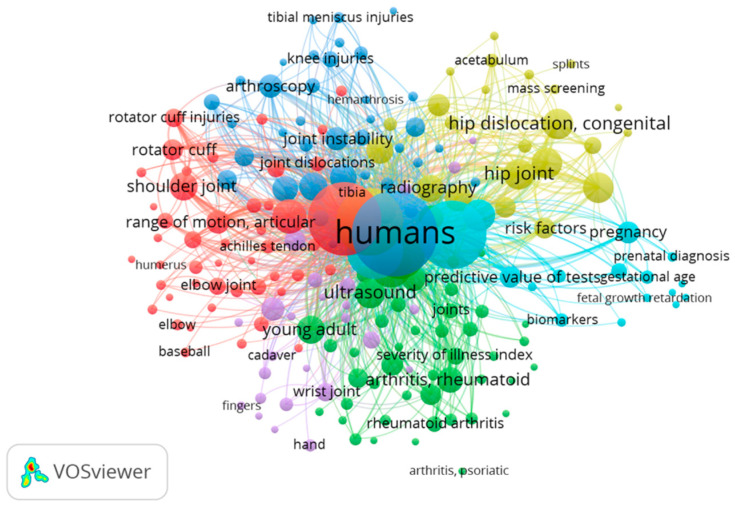
Visualization of keywords for ultrasound examination and joint.

The US examination domains could roughly be divided into 6 modules, corresponding to the six main types of anatomical structures evaluated. In the co-occurence analysis of keywords, we set the minimum repetition of frequency to five times. Figure 4 shows the map for “ultrasound examination” AND “joint” that includes 535 papers from 1970 to 2022. Of 1888 words, a total of 200 met the standard. They were characterized into six clusters. The keywords with the highest frequency in different clusters were: (1) shoulder joint, elbow joint, range of motion, joint dislocations, bursitis and athletes (red); (2) rheumatoid arthritis, cross-sectional studies, hand joints, synovitis and metacarpopahalangeal joints (green); (3) knee joint, knee injuries, ankle joint, ankle injuries and differential diagnosis (blue); (4) hip joint, acetabulum, infant, child, neonatal screening and follow-up studies (yellow); (5) joint diseases, osteoarthritis, wrist joint, wrist diseases and pain (light purple); (6) adult, female, pregnancy, prospective studies and predictive values (light blue). 

**Figure 5:**
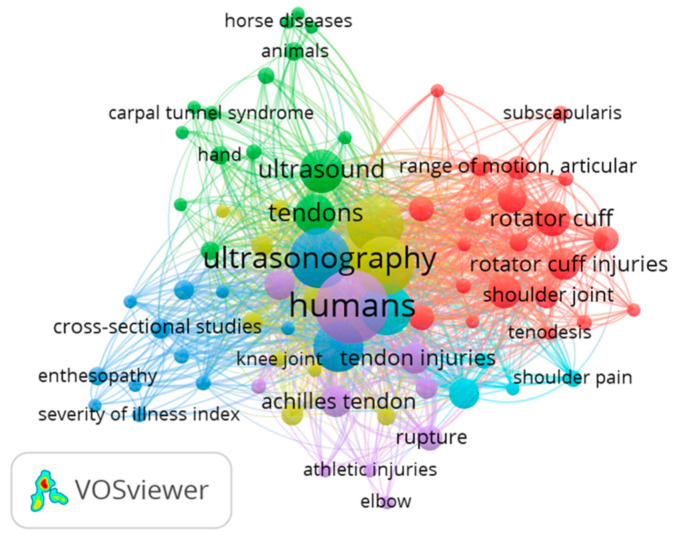
Visualization of keywords for ultrasound examination and tendon.

Figure 5 shows the map for “ultrasound examination” AND “tendon” that include 356 papers from 1970 to 2022. Of 875 words, a total of 76 met the standard. They were characterized into six clusters. The keywords with the highest frequency in different clusters were: (1) rotator cuff, rotator cuff injuries, tenodesis, retrospective studies and shoulder (red); (2) tenosynovitis, hand, carpal tunnel syndrome, patellar ligament and wrist (green); (3) entesopathy, rheumatoid arthritis, physical examination, psoriasis and severity of illness index (blue); (4) male, female, reproducibility of results, aged, adolescent and young adult (yellow); (5) Achilles tendon, tendinopathy, tendon injuries, athletic injuries and rupture (light purple); (6) pain measurements, middle-aged and prospective studies (light blue). 

**Figure 6:**
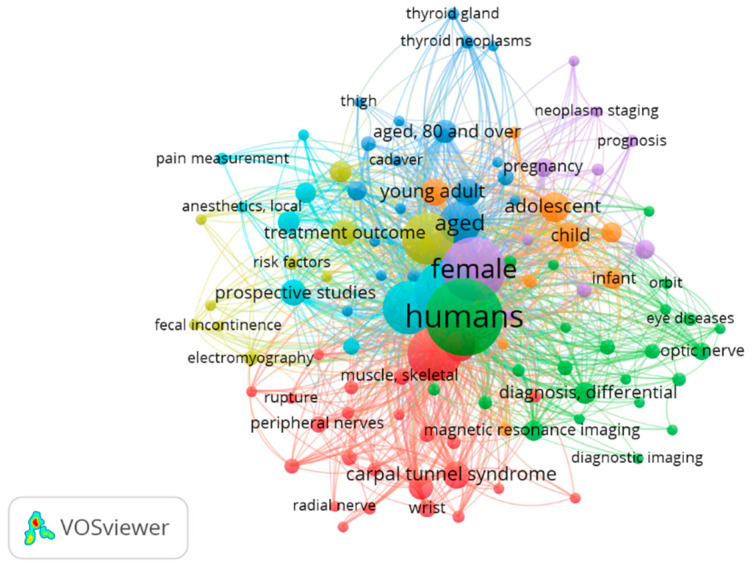
Visualization of keywords for ultrasound examination and nerve.

Regarding “ultrasound Examination” and “nerve”, Figure 6 shows a map that includes 346 papers from 1970 to 2022. Of 1493 words, a total of 107 met the standard. They were characterized into six clusters. The keywords with the highest frequency in different clusters were: (1) carpal tunnel syndrome, median nerve, radial nerve, peripheral nerves and diagnosis (red); (2) differential diagnosis, nerve sheath neoplasms, optic nerve diseases, tibial nerve and neurilemmoma (green); (3) retrospective studies, sciatic nerve, time factors, peroneal nerve and postoperative complications (blue); (4) treatment outcome, electromyography, fecal incontinence, follow-up studies, middle-aged and risk factors (yellow); (5) case–control studies, female and neoplasms staging (light purple); (6) brachial plexus, pain measurement, local anesthetics, prospective studies and nerve block (light blue). 

**Figure 7:**
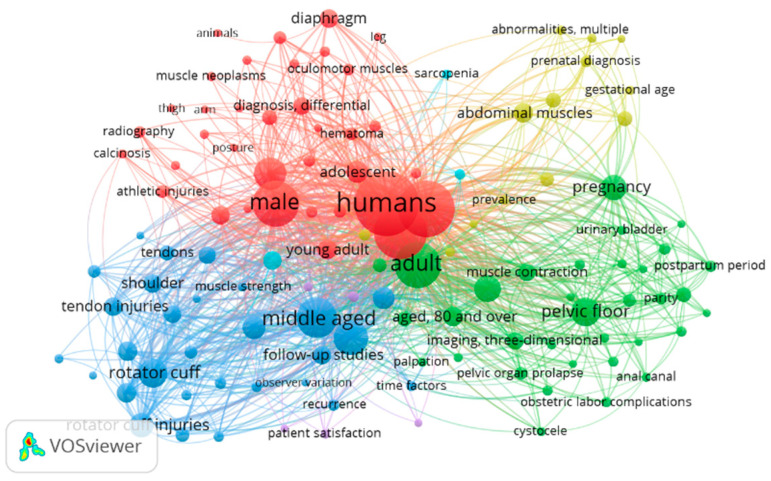
Visualization of keywords for ultrasound examination and skeletal muscle.

Figure 7 shows the map for “ultrasound examination” AND “skeletal muscle” map that includes 309 papers from 1970 to 2022. Of 1244 words, a total of 120 met the standard. They were characterized into six clusters. The keywords with the highest frequency in different clusters were: (1) young adult, rectus abdmonis, leg, male, hematoma and athletic injuries (red); (2) pelvic floor, pregnancy, muscle contraction, 3D imaging and adult (green); (3) middle-aged, recovery of function, retrospective studies, muscle strength and injuries (blue); (4) abdominal muscle, hernia, infant and gestational age (yellow); (5) survey and questionnaires, severity of illness index and single-blind method (light purple); (6) sarcopenia and cross-sectional studies (light blue). 

**Figure 8:**
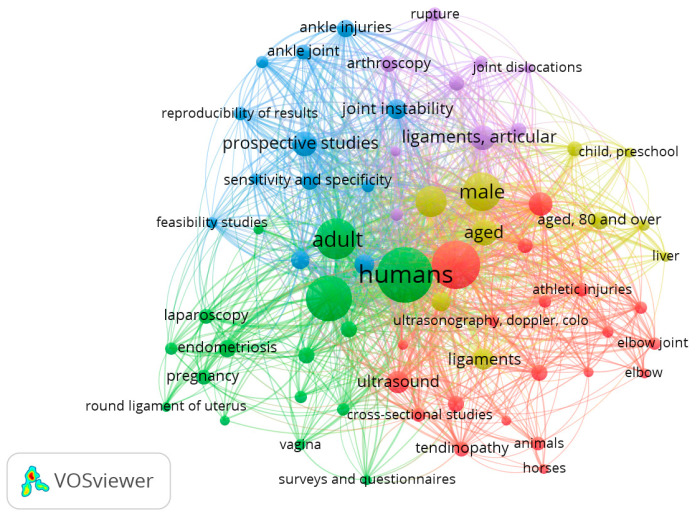
Visualization of keywords for ultrasound examination and ligament.

Regarding “ultrasound Examination” and “ligament”, Figure 8 shows a map that includes 186 papers from 1970 to 2022. Of 760 words, a total of 67 met the standard. They were characterized into five clusters. The keywords with the highest frequency in different clusters were: (1) athletic injuries, collateral ligaments, patellar ligament and knee joint (red); (2) endometriosis, female, adult, pregnancy and treatment outcome (green); (3) ankle injuries, ankle joint, joint instability, prospective studies and sensitivity and specificity (blue); (4) differential diagnosis, child, middle-aged and 80 over aged (yellow); (5) anterior cruciate ligament, rupture and postoperative complications (light purple). 

**Figure 9:**
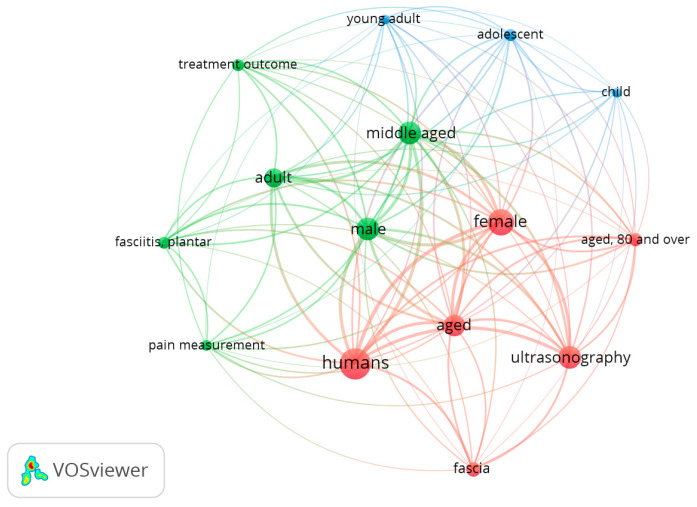
Visualization of keywords for ultrasound examination and fascia.

Finally, Figure 9 reports the map that includes 58 papers from 1970 to 2022. Of 317 words, a total of 15 met the standard. They were characterized into three clusters. The keywords with the highest frequency in different clusters were: (1) aged, female and ultrasonography (red); (2) pain measurement, plantar fasciitis, middle aged and male, (green); (3) adolescent, child and young (blue).

## 4. Discussion

The research related to ‘US examination’ has gained increased interest in the past decade. This study found that the United States ranked first, followed by Italy, as regards the new terminology. Likewise, the *Journal of Ultrasound in Medicine* seems to be the most influential journal in the field, introducing/using this term. Overall, the number of articles related to US examination of the musculoskeletal system shows a growing trend. This would definitely be related to the mounting use of US imaging among physicians of different specialties. In other words, the more they use it, the more they realize how efficient/effective it could be.

Detailed analysis of keyword co-occurrence results helps us to grasp the research hotspots of US examination of the musculoskeletal system for the future. All anatomical structures were assessed in this study with particular emphasis on the assessment of pain, range of motion, interventions and treatment outcomes, in particular for topics as tendons of shoulder joint and tendinopathy. This is in agreement with the finding that the shoulder is the most frequently examined body part using musculoskeletal US, usually for rotator cuff disease [22]. Regarding the evaluation of muscles, in particular in the lower extremity, the data underline the important role of US examination of these structure during muscle contraction in the planning of various exercises. Concerning joints, the research direction is mainly focused on biomechanical phenomena. The ankle, knee and elbow are joints belonging to anatomical regions that suffer problems of instability [8]. Studies have manifested that these phenomena can be highlighted by dynamic US evaluation [23,24,25,26].

US examination of the musculoskeletal system differs (anatomically and histologically) between children [27], adults and elderly people. For instance, while the main goal of US examination in newborns is perhaps to assess the hip joint for congenital hip dysplasia, in elderly people it is to evaluate the body composition for diagnosing sarcopenia [28,29]. The findings of this analysis showed the presence of gender and age medicine, highlightable with the presence of a dedicated cluster for all anatomical structures.

One notable drawback of US imaging is its operator-dependency, i.e., the quality and consistency of US examination rely on the expertise of the examiner [19]. Herewith, this is also the same for physical examination. Therefore, US training requires a long learning curve and dedicated time for the training, particularly for beginners [19]. In the literature, many authors have already proposed the progressive incorporation of US skills in medical school curricula [20] and during residency [21,30]. All such attempts are thought to provide better awareness as regards the practical application of US examination as a natural and spontaneous link between clinical anatomy, diagnosis and management of the patient [9]. Ironically, the ‘operator-dependency’ can also be perceived in a favorable way, yet the interaction between the examiner and the patient is invaluable. Additional clinical history about the precise location and character of symptoms, direct feedback about tenderness with probe palpation, and positions/movements that elicit or aggravate symptoms can all assist in the accurate interpretation of clinical findings. 

As a limitation, this study only searched articles in English, which may lead to some bias; moreover, about countries and regions, the analysis takes into account only the first author. However, to our best knowledge, this is the first paper to investigate the status and future hotspots of the term ‘US examination’ of the musculoskeletal system. 

## 5. Conclusions

In a nutshell, the use of the term “US examination” of the musculoskeletal system seems to increase in the literature. The growing utility of US in daily clinical practice and increased awareness of physicians’ havenatural/spontaneous contribution in this sense. Needless to say, “fusion” of classical physical examination and static/dynamic US imaging has the potential to further patient management as well as research in musculoskeletal practice.

## Figures and Tables

**Table 1:** Top six journals that published on ‘ultrasound examination’ of the musculoskeletal system. Total number of journals that published on ‘ultrasound examination’ of the musculoskeletal system = 160 journals.

Journals	Publications (N)
Journal of Ultrasound in Medicine	26
Ultrasound in Medicine and Biology	23
American Journal of Physical and Rehabilitation Medicine Clinical and Experimental Rheumatology	21 18
Annals of the Rheumatic Diseases	14
Arthritis Care and Research	14

## Data Availability

The data presented in this study are available on request from the corresponding author. The data are not publicly available due to privacy.
